# Vitexicarpin suppresses colorectal and non-small cell lung cancer via selective inhibition of Anoctamin 1

**DOI:** 10.3389/fphar.2025.1557193

**Published:** 2025-05-30

**Authors:** Yohan Seo, Sion Lee, Raju Das, Sung Baek Jeong, Chul Soon Park, Minuk Kim, Deok Kyu Yoon, Armin Sultana, Kantu Das, Jae-Eon Lee, Yong Hyun Jeon, Phan Thi Thanh Huong, Nguyen Xuan Nhiem, Joohan Woo

**Affiliations:** ^1^ Department of Bio-nanomaterials, Bio Campus of Korea Polytechnics, Nonsan, Republic of Korea; ^2^ New Drug Development Center, Daegu-Gyeongbuk Medical Innovation Foundation (KMEDIhub), Daegu, Republic of Korea; ^3^ Department of Physiology, Dongguk University College of Medicine, Gyeongju, Republic of Korea; ^4^ Department of Medical Device Development Center, Daegu-Gyeongbuk Medical Innovation Foundation (KMEDI hub), Daegu, Republic of Korea; ^5^ Department of Computer Science, Southern University Bangladesh, Chittagong, Bangladesh; ^6^ Preclincial Research Center (PRC), Daegu-Gyeongbuk Medical Innovation Foundation (K-MEDI hub), Daegu, Republic of Korea; ^7^ Institute of Marine and Biochemistry, Vietnam Academy of Science and Technology (VAST), Hanoi, Vietnam; ^8^ Channelopathy Research Center (CRC), Dongguk University College of Medicine, Goyang, Gyeonggi-do, Republic of Korea

**Keywords:** ANO1, vitexicarpin, colorectal cancer, non-small cell lung cancer, apoptosis

## Abstract

Colorectal cancer (CRC) and non-small cell lung cancer (NSCLC) remain among the most challenging malignancies to treat due to therapy complexity, adverse events, and dose-limiting toxicities, which often result in treatment failure. NSCLC, in particular, has a high mortality rate attributed to late-stage diagnosis and therapeutic resistance. Anoctamin 1 (ANO1), a calcium-activated chloride channel, has been implicated in cancer progression and is an emerging therapeutic target. In this study, we identified vitexicarpin, a flavonoid isolated from Vitex trifolia, as a novel ANO1 inhibitor with anticancer potential. Vitexicarpin inhibited ANO1 channel function, reduced ANO1 protein levels, decreased cancer cell viability, and induced apoptosis in CRC and NSCLC cell lines. Importantly, vitexicarpin exhibited minimal hepatotoxicity and negligible hERG channel inhibition, supporting its safety profile. Collectively, our findings suggest that vitexicarpin is a promising candidate for the treatment of CRC and NSCLC through selective inhibition of ANO1.

## Introduction

Colorectal cancer (CRC) and non-small cell lung cancer (NSCLC) are among the leading causes of cancer-related mortality worldwide. Advances in surgery, radiotherapy, chemotherapy, targeted therapy, and immunotherapy have improved outcomes; however, treatment complexity, adverse events, and emerging resistance often compromise efficacy (Ciardiello et al., 2022; Zielińska et al., 2021; Duma et al., 2019; Bourreau et al., 2023; Miller et al., 2022).

Anoctamin 1 (ANO1), also known as transmembrane protein 16A (TMEM16A), is a calcium-activated chloride channel that has been implicated in tumorigenesis and cancer progression (Ji et al., 2019). Pharmacological inhibition of ANO1 has demonstrated anticancer effects in head and neck squamous cell carcinoma (HNSCC), esophageal squamous cell carcinoma (ESCC), gastrointestinal stromal tumors, CRC, and NSCLC (Liu et al., 2021; Zhong et al., 2021). Inhibiting ANO1 not only reduces cell viability but also impairs cell migration and proliferation across multiple cancer types (Hao et al., 2021; Ji et al., 2019).

Several ANO1 inhibitors have been identified, including CaCCinh-A01, luteolin, idebenone, Ani9, and schisandrathera D (Jeong et al., 2022; Liu et al., 2015; Park et al., 2023; Seo et al., 2015; 2016; 2017; 2020a; b, 2021). However, many of these compounds exhibit limited potency or specificity toward ANO1, underscoring the need for more selective and effective inhibitors.

Vitex trifolia, a coastal shrub traditionally used to treat inflammation, liver disorders, and tumors, is rich in bioactive flavonoids such as vitexicarpin (casticin), persicogenin, and penduletin (Chan et al., 2018; Vo et al., 2022; You et al., 1998; Adebesin et al., 2022). Vitexicarpin has demonstrated anticancer activities through induction of apoptosis and cell cycle arrest, yet its precise molecular mechanism, particularly in relation to ion channel regulation, remains poorly understood.

In this study, we isolated vitexicarpin from V. trifolia and evaluated its effects on ANO1 channel function and protein expression in CRC and NSCLC cells. Our findings reveal that vitexicarpin is a novel ANO1 inhibitor with promising anticancer potential.

## Materials and methods

### Plant material

The fruits of *V. trifolia* were collected from the Xuan Thuy National Garden, Nam Dinh, Vietnam, in October 2021. Botanical identification was performed by Dr. Nguyen The Cuong of the Institute of Ecology and Biological Resources, Vietnam Academy of Science and Technology (VAST). A voucher specimen (No. NCCT-P133) was deposited at the Institute of Chemistry, VAST.

### Extraction and isolation

Dried *V. trifolia* fruit powder (5 kg) was subjected to extraction using 100% MeOH with sonication (thrice, each time with 15 L MeOH). After solvent evaporation, the resulting MeOH extract (350 g) was reconstituted in water and partitioned using *n*-hexane (H), CH_2_Cl_2_ (D), and EtOAc (E) to yield H (30 g), D (14 g), and E (14 g) and an aqueous layer (70 g). Fraction D was chromatographed on silica gel using a gradient solvent mixture of hexane and acetone to yield four subfractions (D1–D4). Subsequently, fraction D2 (3 g) was subjected to chromatography on a YMC RP-18 column (YMC Co., Ltd., Kyoto, Japan) using a 1:1 v/v mixture of acetone and water, resulting in the isolation of D2E5 (126 mg). This subfraction was further purified by prep-HPLC using a J’sphere ODS M80 column (250 mm × 20 mm; YMC Co., Ltd.) and a mobile phase of 38% aqueous acetonitrile at a flow rate of 3 mL/min to isolate vitexicarpin (casticin: 3′,5-dihydroxy-3,4′,6,7-tetramethoxyflavone, 12 mg). The chemical structure was determined using NMR spectroscopy, and the NMR data were compared with those reported in literature (Vo et al., 2022).

### Cell culture

PC9 (NSCLC), PC3 (prostate cancer), and HT29 (colorectal cancer) cells were cultured in RPMI 1640 supplemented with 10% fetal bovine serum (FBS), 2 mM L-glutamine, 100 U/mL penicillin, and 100 μg/mL streptomycin. Fischer rat thyroid (FRT) cells were maintained in DMEM with the same supplements. All cells were incubated at 37°C in a humidified 5% CO_2_.

### Yellow fluorescent protein fluorescence quenching analysis

PC9 cells stably expressing the YFP variant (YFP-H148Q/I152L/F46L) and endogenous ANO1 were seeded in 96-well plates at a density of 5 × 10^3^ cells/well. After 48 h of incubation, the cells were washed twice with phosphate-buffered saline and incubated for 10 min with the test compounds dissolved in phosphate-buffered saline. YFP fluorescence was measured every 0.4 s for 5 s using a FLUOstar^®^ Omega microplate reader (BMG Labtech, Ortenberg, Germany). ANO1-mediated iodide influx was measured 1 s after injecting 100 μL of 70 mM iodide solution with 100 μM ATP into each well. The inhibitory effects of the test compounds on ANO1 activity were assessed based on the initial iodide influx rate, which was calculated from the initial slope of the decrease in fluorescence after ATP injection.

### Short-circuit current

Snapwell inserts containing ANO1 or CFTR-expressing FRT cells were mounted in Ussing chambers (Physiologic Instruments, San Diego, CA, USA), and the apical and basolateral baths were filled with an HCO_3_-buffered solution containing 120 mM NaCl, 5 mM KCl, 1 mM MgCl_2_,1 mM CaCl_2_, 10 mM d-glucose, 2.5 mM HEPES, and 25 mM NaHCO_3_ (pH 7.4). For the FRT cells, the basolateral bath was filled with an HCO_3_-buffered solution, and the apical bath was filled with a half-Cl solution. In the half-Cl solution, 65 mM NaCl in the HCO_3_-buffered solution was replaced with sodium gluconate. The basolateral membrane was permeabilized with 250 μg/mL amphotericin B, after which the cells were bathed for 20 min to stabilize and aerated with 95% O_2_/5% CO_2_ at 37°C. Thereafter, forskolin was applied to the apical bath solution to induce an intracellular cyclicAMP increase. Subsequently, vitexicarpin and CFTR_inh_-172 were added to the apical and basolateral bath solutions. The apical membrane currents were measured using an EVC4000 Multi-Channel V/I Clamp (World Precision Instruments, Sarasota, FL, USA) and PowerLab 4/35 software (AD Instruments, Castle Hill, NSW, Australia). Data were analyzed using LabChart Pro 7 (AD Instruments). The sampling rate was 4 Hz.

### Measurement of intracellular calcium levels

PC9 cells were cultured in black-walled 96-well microplates and loaded with Fluo-4 NW calcium indicators (Invitrogen, Carlsbad, CA, USA) according to the manufacturer’s protocol. After 1 h of incubation, the plates were transferred to a FLUOstar^®^ Omega microplate reader (BMG Labtech) with custom Fluo-4 excitation/emission filters (485/538 nm) to measure Fluo-4 fluorescence.

### Cell line generation and characterization

PC9 cells were cultured in RPMI-1640 medium supplemented with 10% FBS and antibiotics under standard conditions (37°C, 5% CO2). To initiate selection, cells were exposed to 50 nM gefitinib, a sub-lethal concentration that partially inhibits proliferation, to allow gradual adaptation. Over a period of 3 to 6 months, the gefitinib concentration was incrementally increased every 1–2 weeks, depending on cell viability and confluence. The typical escalation followed this nM → 100 nM → 200 nM → 500 nM → 1 µM → 2 µM → 5 µM → 10 µM. At each concentration, cells were allowed to recover and proliferate before progressing to a higher dose. Resistance was confirmed via MTT cell viability assay and dose-response curve analysis, comparing IC50 values between parental PC9 and PC9-GR cells.

### Molecular docking analysis

The selected chemical entities and proteins of interest were essential to rectifying topological perturbation. With this regard, the ligPrep module of the Schrodinger suite was utilized for ligand preparation. The three-dimensional (3D) confirmation of the selected ligand associated with the lowest geometry was achieved by maintaining the default settings. Besides, the appropriate protonation state for each ligand was determined at physiological conditions. Using the all-atom Optimized Potentials for Liquid Simulations (OPLS3) force field, 32 conformers per ligand were minimized before the final docking (Meng et al., 2011).

The first ligand-bound ANO1 receptor (PDB ID: 7ZK3) belongs to the *Mus musculus* organism, appeared in the protein data bank and has been considered for molecular docking and dynamics simulation. Topologically, ANO1 is a homodimer; therefore, only Chain A was counted to simplify the calculation. The proper bond orders, appropriate hydrogen, and disulfide bonds were assigned to the structure. Subsequently, all irrelevant water molecules from het groups were deleted, and missing side chains and loops were filled by prime. The protonated states were also confirmed at pH 7.0. To refine the final structure, the network of H-bond was optimized, and energy minimization was processed with the OPLS3 force field, where the converge heavy atoms were restrained to RMSD 0.30Å.

At the initial stage, the re-docking module was implemented so that the docking efficiency could be evaluated, as well as false positive findings could be determined. After superimposing the re-docked and co-crystallized complex, the root mean square deviation (RMSD) was determined to be less than 2Å. In general, Glide has three distinct levels of scoring functions, specifically Glide-HTVS, Glide-SP (Standard Precision), and Glide-XP (Extra Precision). The scoring functions incorporate the Emodel scoring function, which is essentially composed of the Glide-score and protein-ligand coulomb-vdW energy components. We used Glide-XP docking, with a focus on ligand sampling conducted in a flexible manner. The scaling factor and partial charge for the nonpolar components of the ligand were designated as 0.80 and 0.15, respectively. Then, final docked complexes with optimal negative energy were chosen for subsequent investigation.

### Prime molecular mechanics with generalized born and surface area solvation (MM-GBSA)

MM-GBSA analysis of the prime module was performed on complexes formed by the docking simulation. Using the OPLS3 molecular mechanics force field, MM-GBSA (Mark and Nilsson, 2001) calculates the relative binding energy by combining the molecular mechanics energy (EMM), SGB solvation model for polar solvation (GSGB), and non-polar solvation term (GNP) composed of a non-polar solvent-accessible surface area and van der Waals interactions.

The total free energy of binding is calculated as follows:

$${\Delta G}_{bind}{=G}_{complex}-\left(G_{protein}{+G}_{ligand}\right)$$

where G = EMM + GSGB + GNP.

### Molecular dynamic simulation

The protein-ligand complex with the most favourable binding pose was subjected to molecular dynamics simulation studies to check the structure stability of selected complexes. A total of 100 ns classical MD simulation runs for each complex were executed in the Desmond academic platform. All systems were prepared and minimized by the system builder. An orthorhombic simulation box was selected for maintaining periodic boundary conditions at 10Å of each direction and filled with the TIP3P water model (Mark and Nilsson, 2001). The total system charge was neutralized by adding opposite counter ions, and then 0.15M salt concentration was maintained by adding NaCl salt to maintain physiological conditions. The steepest descent algorithms were followed to minimize the system. The Short-range Coulombic interactions were assessed at a cutoff radius of 9Å, while long-range electrostatic interactions were determined using the particle mesh Ewald method. After reaching the equilibration, an NPT ensemble with a Nose-Hoover chain thermostat was used with 300 K temperature and 1.01325 bar pressure for the final production run, where each trajectory was saved after every 100ps for final analysis. A relevant POPC membrane model was used (Kurki et al., 2022) and the all-atom OPLS forcefield was selected for the whole simulation process.

### Western blot analysis

Protein samples (20–60 μg) were separated using 4%–12% Tris-Glycine-PAG Pre-Cast Gel (Koma Biotech, Seoul, South Korea) and transferred onto polyvinylidene fluoride membranes. The membranes were blocked for 1 h with 5% bovine serum albumin in Tris-buffered saline containing 0.1% Tween 20, after which they were incubated with primary antibodies, including anti-ANO1 (Abcam, Cambridge, UK) and anti-β-actin (Santa Cruz Biotechnology, Dallas, TX, USA), followed by horseradish peroxidase-conjugated anti-secondary IgG antibodies (Enzo Life Sciences, Inc., Farmingdale, NY, USA) for 1 h. Visualization was performed using an ECL Plus Western Blotting System (GE Healthcare, Chicago, IL, USA).

### Cell viability assay

Cell viability was assayed using a Cell Counting Kit CCK -8 (Dojindo, Rockville, MD, USA), and MTS cell viability assay was performed using a CellTiter 96^®^ Aqueous One Solution Cell Proliferation Assay Kit (Promega, Madison, WI, USA). PC3, PC9, and HT29 cells were cultured in 96-well plates for 24 h to approximately 20% confluence, after which vitexicarpin (0.03–300 μM) or vehicle (dimethyl sulfoxide) was added to the medium. After 72 h of treatment, the medium was completely removed, and CCK-8 or MTS assays were performed according to the manufacturer’s instructions. The absorbance of formazan was measured at a wavelength of 490 or 450 nm using a microplate reader (Synergy™ Neo; BioTek, Winooski, VT, USA).

### Caspase-3/CPP32 colorimetric assay

A caspase-3/CPP32 colorimetric assay was performed according to the manufacturer’s instructions (#K106; BioVison, Milpitas, CA, USA). Briefly, HT29 and PC9 cells were cultured in 6-well plates until 70% confluence, and the test compound or Ani9 (an ANO1 inhibitor) was added to the wells. After incubation for 24 h, 5 × 10^6^ cells were lysed in cell lysis buffer for 10 min at 4°C. Subsequently, the cells were centrifuged for 10 min, and the supernatants were collected. Thereafter, 100 μg protein or 50 μL buffer was added to each well with 2× reaction buffer containing 10 mM DTT. To measure caspase-3 activity, 5 μL DEVD-pNA substrate was added and incubated for 1 h at 37°C. The optical density was measured at the wavelength of 400 nm using a microplate reader (Synergy™ Neo).

### Human cleaved PARP-1 activity assay

A human cleaved PARP-1 activity assay was performed according to the manufacturer’s instructions (#ab174441; Abcam). Briefly, HT29 and PC9 cells were cultured in 6-well plates until 80% confluence. Next, the test compounds were added and incubated with the cells for 24 h. Subsequently, 5 × 10^7^ cells were lysed in cell extraction buffer for 20 min and then centrifuged at 13,000 rpm for 20 min at 4°C to collect the supernatant. Next, 100 μg protein or 50 μL buffer was added to an antibody cocktail containing capture and detector antibodies in each well and incubated for 1 h. Afterward, the wells were washed twice with 1× wash buffer, after which TMB development solution was added, and the contents of the wells were incubated for 10 min. Finally, a stop solution was added, and the optical density was measured at a wavelength of 450 nm using a microplate reader (Synergy^TM^ Neo).

### Cell cycle analysis

PC9 and HT29 cells were seeded at 2 × 10^5^ cells/well in 100-phi culture plates and treated with the test compound for 24 h. Next, the cells were fixed with cold 70% ethanol and incubated for 2 h at −20°C. The ethanol was removed, and the cells were resuspended in propidium iodide, Triton X-100, or DNAse-free RNAse A staining solution for 30 min. Cell cycle distribution was analyzed using Gallios (Beckman Coulter, Brea, CA, USA). Approximately 2 × 10^3^ cells per group were analyzed.

### hERG inhibition assay by patch-clamping electrophysiology

To assess the cardiotoxic effects of the tested hERG channel-dependent chemical compounds, we measured the rate of hERG channel inhibition in hERG-overexpressing HEK cells (Eurofins Scientific, Luxembourg) using a PatchLiner automated patch-clamping system (Nanion Technologies, Munich, Germany).

The cells were incubated at 37°C under 5% CO_2_. Subsequently, the cells were detached from the culture plates using trypsin (SH30042.02; HyClone, Logan, UT, USA) and centrifuged at 200× *g*. After washing with an external standard buffer solution (08 3001; Nanion Technologies), the cells were resuspended in 5 mL fresh external standard buffer solution. The resulting cell suspension was loaded onto the PatchLiner system. The cells were then automatically dispensed into the wells of an NPC-16 chip (071102; Nanion Technologies), and one cell per well was sealed in a microhole at the bottom. Once a GΩ seal was formed, light and short suction pulses were applied to break through the membrane and establish a whole-cell mode to electrically connect the cell with the Internal KF110 buffer solution (08 3007; Nanion Technologies).

To generate voltage stimulation specific to the hERG channel, the membrane potential of the cells was initially maintained at −80 mV, followed sequentially by −40 mV (0.5 s), +40 mV (0.5 s), −40 mV (0.5 s), and −80 mV (0.2 s) to generate hERG tail currents. The peak hERG tail current recorded from each well represented the baseline level of hERG channel activity. To investigate the changes in the hERG activity caused by the tested chemical compounds, the relevant chemical stock solutions were diluted with an external standard buffer solution to achieve the desired concentrations and automatically added to the wells of an NPC-16 chip. hERG channel activity was measured under the same voltage conditions. Finally, the relative changes in hERG activity induced by the tested compounds were calculated and expressed as percentages using the following formula:

$$\%hERG\;activity=\left(peak\;hERG\;tail\;current\;after\;compound\;treatment/peak\;hERG\;tail\;current\;before\;compound\;treatment\right)\times 100$$

### Resazurin reduction assay for evaluating hepatocellular viability

Hepatocellular viability was assessed using the resazurin reduction assay. HepG2 hepatocellular carcinoma cells (10,000 cells) were seeded in each well of a black & clear-bottom 96-well plate containing DMEM High Glucose supplemented with 10% (v/v) fetal bovine serum and 100 μg/mL penicillin–streptomycin. Subsequently, the cells were incubated in a 5% CO_2_-supplied 37°C incubator for 15–20 h.

The following day, the cells were treated with the tested compounds at four different concentrations (0, 0.05, 0.5, 5, and 50 μM) using DMEM High Glucose supplemented with 1% (v/v) fetal bovine serum and 100 μg/mL penicillin–streptomycin. For maximum and minimum cell viability control experiments, 0.5% (v/v) dimethyl sulfoxide solvent and 0.01% (v/v) Triton X-100 were used.

After incubation for 20 h, each well was treated with 20% (v/v) resazurin reagent (#G8080; Promega) and further incubated for 2 h. Colorimetric analysis of the resorufin levels in each well was performed by determining the fluorescence intensity at a wavelength of 590 nm. Each data point was normalized using the maximum/minimum cell viability data and presented as “%Relative viability” using the following formula:

$$\%Relative\;viability=\left(experimental\;datum\;–\;minimal\;viability\;datum\right)/\left(maximal\;viability\;datum\;–\;minimal\;viability\;datum\right)\times 100$$

### Real-time RT-PCR analysis

Total mRNA was extracted using TRIzol reagent (Invitrogen, Carlsbad, CA, USA). Complementary DNA was synthesized using random hexamer primers, an oligo(dT) primer, and SuperScript III Reverse Transcriptase (Invitrogen). Quantitative real-time PCR was performed using the StepOnePlus Real-Time PCR System (Applied Biosystems, Foster City, CA, USA) with Thunderbird SYBR qPCR mix (Toyobo, Osaka, Japan). The thermal cycling conditions were as follows: initial denaturation at 95°C for 5 min, followed by 40 cycles of 95°C for 10 s, 55 C for 20 s, and 72°C for 10 s.

The primer sequences for human ANO1 were:

Sense: 5′-GGA​GAA​GCA​GCA​TCT​ATT​TG-3′

Antisense: 5′-GAT​CTC​ATA​GAC​AAT​CGT​GC-3′

### Statistical analysis

All experiments were conducted independently at least three times. The results from multiple trials are presented as mean ± standard error of the mean (SEM). Statistical analyses were performed using Student’s t-test or one-way ANOVA, as appropriate. A p-value less than 0.05 was considered statistically significant. Statistical significance was annotated as follows: p < 0.05 (*), p < 0.01 (**), and p < 0.001 (***). GraphPad Prism software (GraphPad Software, Boston, MA, USA) was used to plot dose-response curves and to calculate IC_50_ values.

## Results

### Cell-based high-throughput screening for identifying a novel natural compound that inhibits the ANO1 channel

**FIGURE 1:**
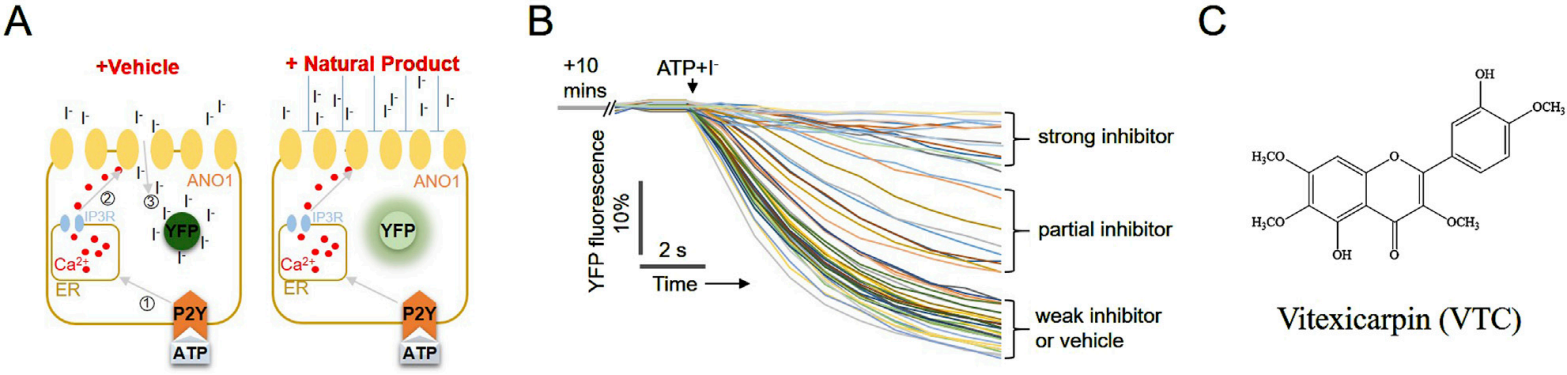
Identification of vitexicarpin using yellow fluorescent protein (YFP)-based high-throughput screening. **(A)** Schematic representation of the cell-based YFP-reduction assay. The activation of the ATP-induced P2Y receptor increases calcium release from the endoplasmic reticulum, thereby activating the ANO1 channel and resulting in an influx of iodide that quenches YFP. **(B)** YFP fluorescence levels correspond to the inhibitory effect of the compounds in cells treated for 10 min. **(C)** Chemical structure of vitexicarpin.

The inhibition of ANO1 by compounds extracted from *V. negundo* has been evaluated using a modified cell-based high-throughput screening system (Jeong et al., 2022). As shown in Figure 1, upon treating cells with ATP, intracellular calcium levels increased, leading to iodine flow into the cell through the ANO1 channel, which also acts as an iodide channel (Park et al., 2023). Intracellular iodine binds to mutant YFP and strongly reduces its fluorescence. However, when an ANO1 inhibitor inhibited the ANO1 channel and blocked iodine influx, no decrease in YFP fluorescence was observed (Figure 1A). To screen for substances isolated from *V. negundo* that exhibit ANO1-inhibiting effects, PC9-YFP cells were treated with natural product compounds for 10 min, and vitexicarpin was selected as a potent inhibitor of ANO1 (Figures 1B,C).

**FIGURE 2:**
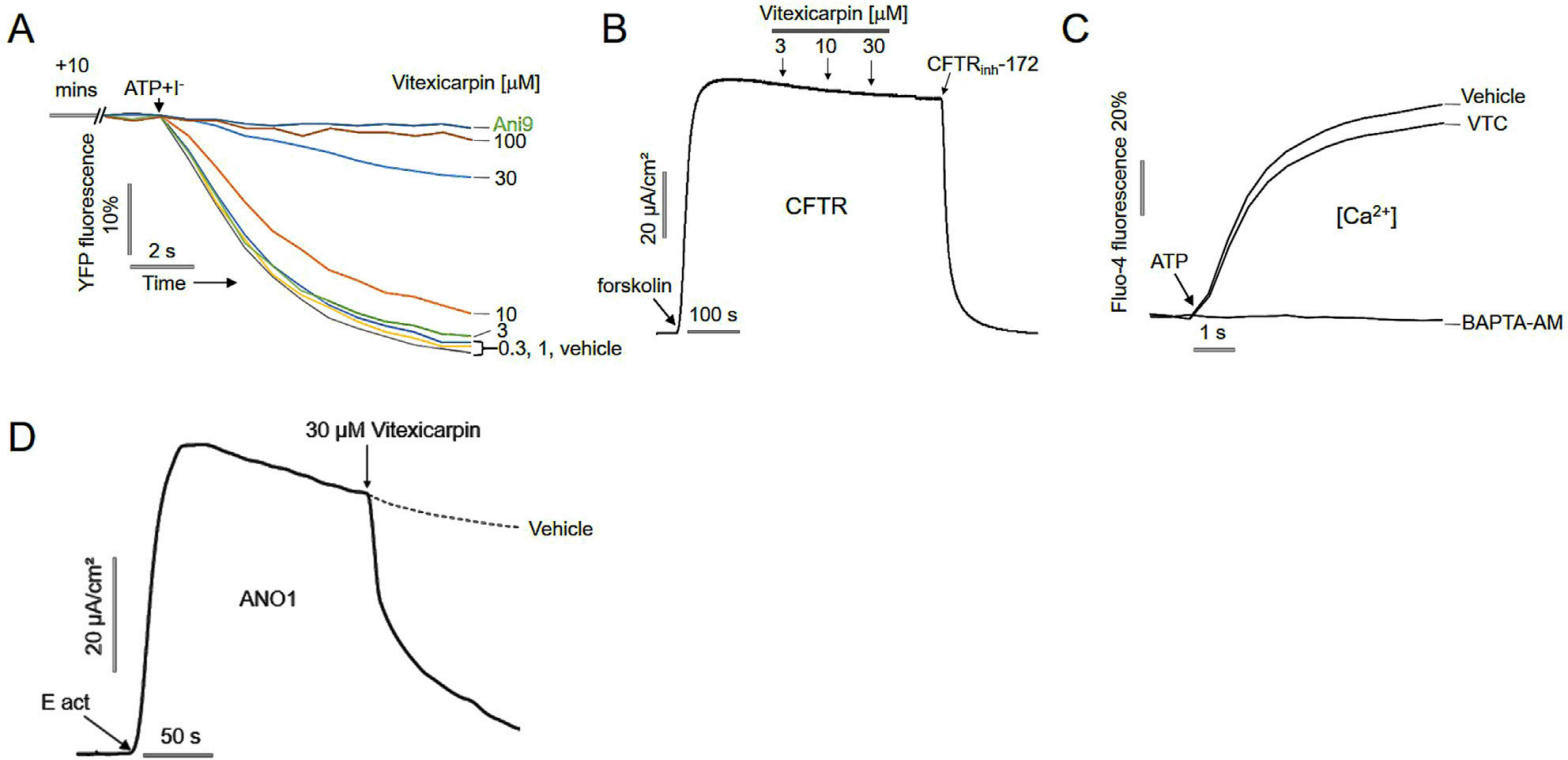
**(A)** Yellow fluorescent protein (YFP) fluorescence levels were measured before treatment with Ani9 and different doses of vitexicarpin. **(B)** Short-circuit current in CFTR- and YFP-expressing FRT cells. Forskolin-induced CFTR activity was inhibited by 10 µM CFTRinh-172 (mean ± SD, *n* = 5). **(C)** Intracellular Ca levels in CHO-K1 cells treated with 30 μM VTC for 20 min, then with 100 μM ATP were measured using Fluo-4 NW (mean ± SD, *n* = 5). **(D)** Short-circuit current measurements showing that vitexicarpin inhibited transepithelial chloride currents in epithelial cells differentiated to express ANO1 (mean ± SD, n = 5).

To verify the inhibitory effect of vitexicarpin on ANO1, FRT cells were treated with Ani9, a selective ANO1 inhibitor. Treatment with Ani9 for 10 min completely blocked YFP fluorescence reduction induced by ANO1 activation, confirming assay validity. Similarly, vitexicarpin inhibited ANO1 activation in a dose-dependent manner (Figure 2A).

To determine whether vitexicarpin selectively inhibits ANO1 without affecting other chloride channels, we evaluated its effect on CFTR channel activity. CFTR-expressing FRT cells were treated with vitexicarpin, and CFTR activation was induced by forskolin stimulation. Vitexicarpin up to 30 µM did not inhibit CFTR-mediated currents, whereas CFTRinh-172, a specific CFTR inhibitor, completely blocked CFTR activity (Figure 2B).

Because ANO1 can be activated at low intracellular calcium concentrations, we examined whether vitexicarpin affected intracellular calcium levels. Treatment with 30 µM vitexicarpin followed by ATP stimulation showed that ATP-induced intracellular calcium elevation was not suppressed by vitexicarpin, while it was completely blocked by BAPTA-AM, a calcium chelator (Figure 2C).

Finally, to confirm the inhibitory effect of vitexicarpin on ANO1 in a physiologically relevant epithelial model, short-circuit current measurements were performed. Vitexicarpin significantly reduced transepithelial chloride currents in epithelial cells differentiated to express ANO1, as measured using the short-circuit current assay system (Figure 2D).

### Effect of vitexicarpin on prediction of binding sites and ANO1 protein levels

ANO1 protein functions as a calcium-activated chloride channel characterized by a unique structure, including two identical subunits enclosing a pore and controlling ion conduction. Each subunit has a hydrophilic cavity surrounded by transmembrane helices TMD3–TMD8. The initial ANO1 structure was postulated to include eight transmembrane domains (TMDs) along with intracellular N- and C-terminal regions (Hawn et al., 2021), although subsequent research provided accurate structural configuration containing ten α-helices that span the transmembrane region. These helices have been observed in bent formations (Brunner et al., 2014). Moreover, the N-terminal domain is involved in intra-subunit interactions with the cytoplasmic C-terminus of the protein. Accumulating evidence suggests that Ca^2+^ modulates ANO1 activity directly. In contrast, membrane lipid PIP_2_ regulates ANO1 activation. Mutagenesis revealed that the acidic residues E654, E702, E705, E734, and D738 are involved in Ca^2+^ binding, thus directly regulating channel opening (Greenwood et al., 2001). In addition, several chemicals have been proposed to bind to the flexible loop region near the extracellular pore entry; however, the exact location of binding and activity is unknown.

### Molecular docking analysis

To ensure the accuracy and reliability of docking outcomes, the re-docking results were analyzed by comparing the root mean square deviation (RMSD) of the re-docked complex with the ligand-bound co-crystalized complex, where an RMSD <2 Å ensured docking accuracy. After the final outcome, vitexicarpin and Ani9 displayed the highest negative docking scores of −6.675 and −6.686 kcal/mol, respectively, relative to that of Eact (−5.339 kcal/mol). The overall docking and MM-GBSA scores are presented in Supplementary Table S1A.

**FIGURE 3:**
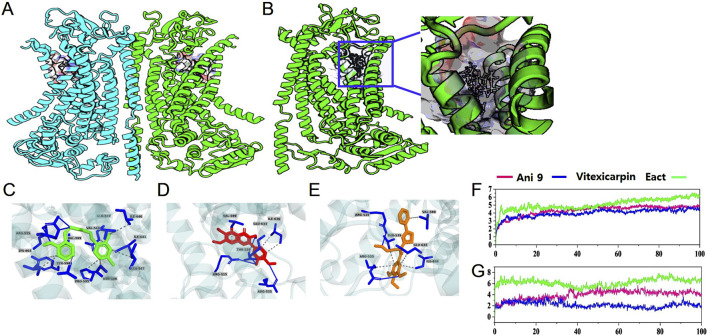
Graphical representation of **(A)** ANO1 structure—homodimeric structure of ANO1. **(B)** Molecular docking at the 1PBC binding site. **(C)** The intermolecular interactions of vitexicarpin, **(D)** Ani9, and **(E)** Eact. **(F,G)** Protein and ligand root mean square deviations of vitexicarpin, Ani9, and Eact.

Regarding protein–ligand binding, intermolecular interactions contribute to the stability of the complex, which is maintained by hydrogen bonds, van der Waals forces, and carbon–hydrogen bonds to a great extent. In this regard, non-bonded protein–ligand complex interactions were considered based on the XP docking pose. The data are presented in Figure 3, which show the participation of vitexicarpin in several interactions with key residues reported in the 1PBC-bound ANO1 structure. Vitexicarpin exhibited hydrogen bonding interactions with residues Arg 515 and Arg 535 and hydrophobic interactions with residues Val 599 and Ile 636 (Figure 3C). Moreover, vitexicarpin interacted with residues Glu 633 and Thr 539 via hydrogen bonding and hydrophobic contact, respectively. Incidentally, the inhibitor 1PBC is also reportedly involved in hydrophobic interactions with residues Val 599 and Ile 636 (Lam et al., 2022 #281). In addition, 1PBC interacts with the non-polar charge residues Thr 539 and Arg 515, both of which are present in the vitexicarpin-ANO1 complex. Thus, similar results regarding the interaction of vitexicarpin with ANO1 at the 1PBC binding site suggest similar outcomes.

To more precisely compare and validate the vitexicarpin docking results, the interaction between the known inhibitor Ani9 and ANO1 was also analyzed, and Ani9 exhibited similar results. Ani9 was stabilized in the binding core by creating a pair of hydrogen bonds with Arg 515 and a single bond with Lys 603 (Figure 3D) (Das et al., 2023). Furthermore, Ani9 established several hydrophobic interactions with the amino acid residues Val 511, Ile 512, Val 543, Asn 546, Leu 547, Pro 595, Tyr 598, Val 599, Gln 637, Ile 640, and Ile 641. Eact, an activator of ANO1, interacted with numerous ANO1 residues at its extracellular location via hydrogen bonding and hydrophobic interactions (Figure 3E). Supplementary Table S1B in the “Supplementary Material” summarizes the molecular interactions.

The each system was simulated for 100ns to evaluate the conformational stability and structural changes of protein upon ligand binding during the simulation period. A low RMSD value indicates higher stability, while a high RMSD value suggests less stability. As shown in Figure 3F, protein RMSD analysis revealed that vitexicarpin and Ani9 achieved equilibrium after 20 ns and remained stable throughout the simulation. In contrast, the activator Eact exhibited fluctuations and higher RMSD for first 20 ns but finally achieved stability until the end of the simulation. Thus, the global RMSD results for the proteins indicated that no major conformational changes occurred during the entire simulation period. The total protein RMSD results are displayed in Figure 3F. Figure 3G shows the overall ligand RMSD, where vitexicarpin and Ani9 reached equilibrium after 40 ns, after which no major fluctuations were observed. Vitexicarpin showed the lowest RMSD value, suggesting higher stability in the ANO1 binding pocket than that of Ani9 and Eact. Based on the ligand RMSD analysis results, all compounds remained stable during the 40-ns period. Notably, vitexicarpin exhibited a lower RMSD value than the other compounds, indicating higher ANO1 binding core stability.

Total protein–ligand contacts and the residual contribution in binding mechanisms are depicted in Supplementary Figure S2. The residues Arg 515, Ile 534, Arg 535, Val 536, Val 543, Glu 633, and Ile 636 play a major role as they predominantly contribute to stabilizing the ligand within the ANO1 binding core. Therefore, it can be argued that these residues play crucial roles in stabilizing vitexicarpin. In Supplementary Figure S2B, the plot on the right shows the number of contacts and their density, where darker orange indicates more than one contact on a frame. Since these residues have also been reported in the structure of 1PBC-bound ANO1, they evidently play a significant role in stabilizing vitexicarpin throughout the simulation (Lam et al., 2022). According to the structure of 1PBC bound to ANO1, Lys 603 is responsible for determining the efficacy of 1PBC. However, we did not observe any interaction between vitexicarpin and Lys 603. The simulated timeframes also revealed that Ani9 had a robust interaction with Lys 603. Additionally, Ani9 interacted extensively with Ala 542, Val 543, and Ile 641. However, unlike 1PBC, Ani9, and vitexicarpin, Eact exhibited strong interaction with Val 538 and Gly 628. Compared with the 1PBC-bound structure, only residues Val 536, Val 543, Arg 515, and Glu 633 interacted with ANO1, although their impact was negligible relative to the total simulation period.

**FIGURE 4:**
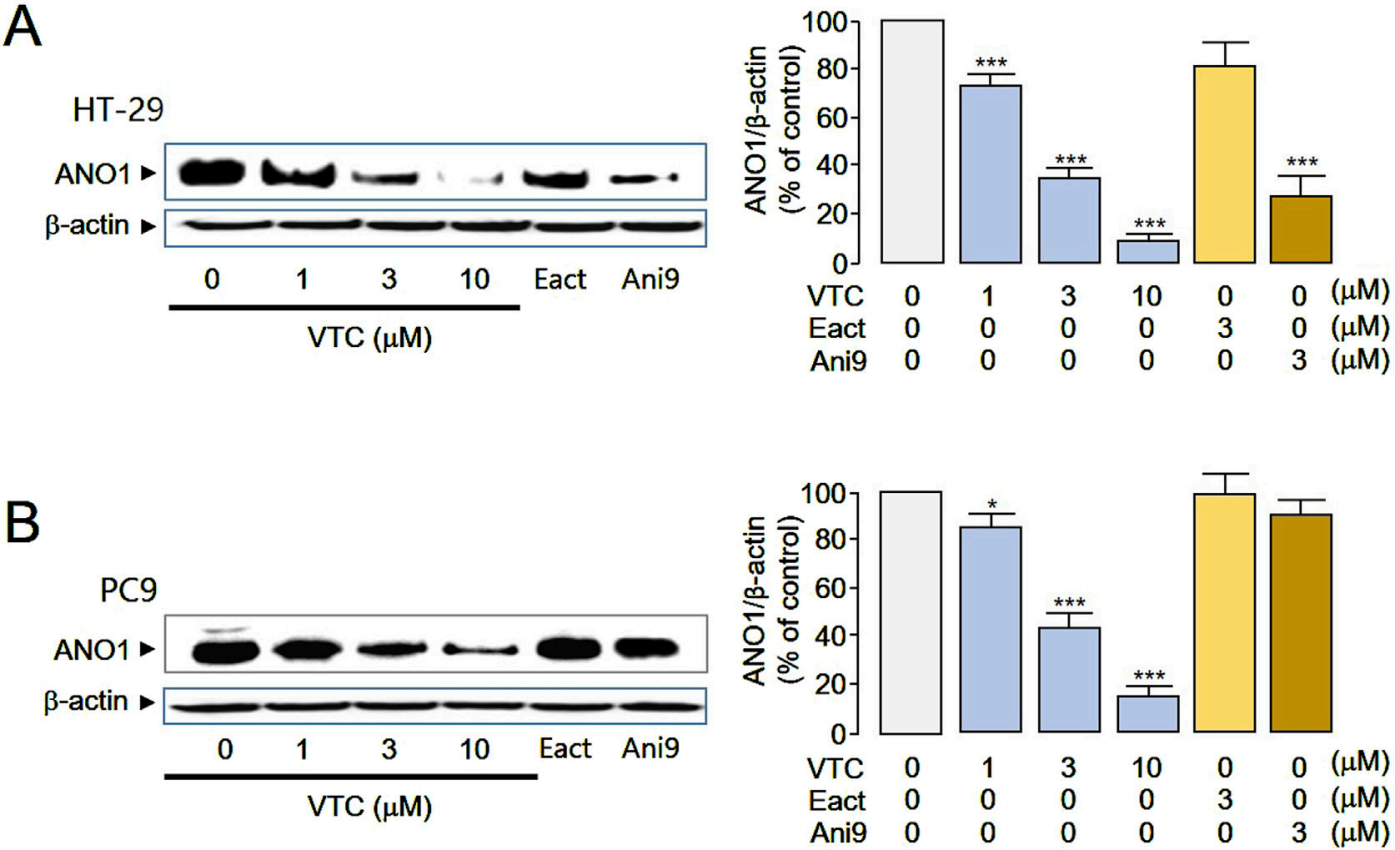
Vitexicarpin reduces ANO1 protein levels in HT29 and PC9 cells. **(A)** HT29 cells were treated with the indicated concentrations of vitexicarpin, Eact, and Ani9 for 24 h, after which ANO1 protein and β-actin levels were measured through western blotting. **(B)** PC9 cells were treated with the indicated concentrations of vitexicarpin, Eact, and Ani9 for 24 h, after which ANO1 protein and β-actin levels were measured through western blotting. (mean ± SD, *n* = 4–5). **p < 0.01., ***p < 0.001.

To confirm whether vitexicarpin reduces ANO1 protein levels based on the docking simulation results, vitexicarpin was added at different concentrations, and changes in ANO1 protein were measured (Figure 4). In HT29 and PC9 cells, vitexicarpin decreased ANO1 protein at different concentrations, and Ani9 decreased ANO1 protein in HT29 cells (Figure 4A) but not in PC9 cells (Figure 4B). Moreover, Eact (an ANO1 functional activator) did not reduce ANO1 protein levels in either cancer cell line (Figure 4). Vitexicarpin appears to downregulate ANO1 at the protein level. To address this, we conducted qRT-PCR analysis and observed no significant change in ANO1 mRNA expression, suggesting that the reduction is not transcriptionally regulated. We hypothesize that Vitexicarpin may promote proteasomal degradation of ANO1. To support this, we have now included proteasome inhibition experiments using MG132 (Supplementary Figure S4), which show restoration of ANO1 levels upon co-treatment, indicating degradation as a likely mechanism.

### Effect of vitexicarpin on viability in gefitinib-resistant cells

**FIGURE 5:**
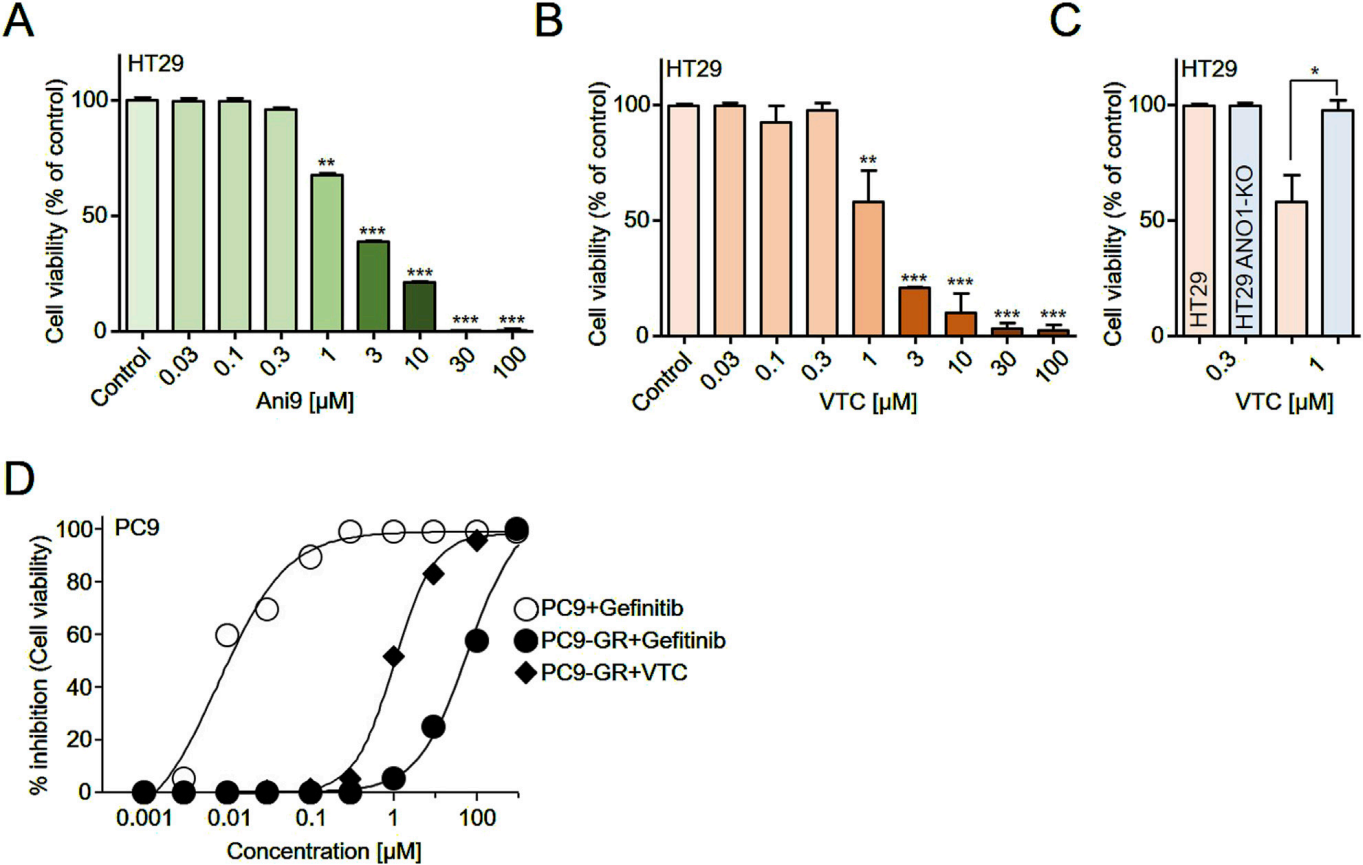
Effects of vitexicarpin on cell viability. **(A,B)** HT29 cells were treated with increasing concentrations (0.03–100 μM) of Ani9 or vitexicarpin for 72 h, and cell viability was measured using a CCK-8 assay. **(C)** HT29 wild-type and HT29 ANO1 knockout (KO) cells were treated with 0.3 and 1 μM vitexicarpin for 72 h, and cell viability was compared using a CCK-8 assay. **(D)** PC9 and gefitinib-resistant PC9 cells (PC9-GR) were treated with increasing concentrations (0.001–300 μM) of vitexicarpin and gefitinib for 72 h, and cell viability was assessed using a CCK-8 assay. Data are presented as mean ± SD (n = 5). Statistical significance was determined by Student’s unpaired t-test. *p < 0.05, **p < 0.01, ***p < 0.001.

The pharmacological inhibition of ANO1 has been reported to suppress the growth of various cancer cell types (Seo et al., 2016; Seo et al., 2020a; b). Given that both Ani9 and vitexicarpin reduced ANO1 protein expression, we investigated whether these compounds inhibit colorectal cancer cell growth through ANO1 downregulation (Figure 5). Consistent with this hypothesis, treatment with Ani9 and vitexicarpin resulted in a dose-dependent decrease in HT29 cell viability (Figures 5A,B), as well as in PC3 cells (Supplementary Figure S2).

To further validate the role of ANO1, we compared the effects of vitexicarpin in HT29 wild-type and ANO1 knockout (KO) cells. Notably, the inhibitory effect of vitexicarpin on cell viability was significantly attenuated in ANO1 KO cells, suggesting that ANO1 is a critical target mediating vitexicarpin’s anticancer activity (Figure 5C).

In the context of non-small cell lung cancer (NSCLC) therapy, gefitinib is widely used; however, 40%–50% of patients eventually develop resistance. To model this clinical challenge, we established a gefitinib-resistant PC9 cell line (PC9-GR) and assessed whether vitexicarpin could overcome resistance. Treatment with gefitinib led to a strong, dose-dependent inhibition of PC9 cell viability, with an IC_50_ of 7.9 nM, while the gefitinib-resistant PC9-GR cells showed an IC_50_ of approximately 7.8 μM (7,800 nM). This represents a dramatic ∼987-fold increase in IC_50_, clearly indicating the acquisition of strong resistance to gefitinibWe subsequently tested vitexicarpin in the resistant PC9-GR cells and observed that it retained significant anticancer activity with an IC50 of approximately 1.01 μM. (Figure 5D).

Collectively, these findings demonstrate that vitexicarpin not only suppresses colorectal cancer cell growth through ANO1 downregulation but also effectively overcomes gefitinib resistance in NSCLC cells.

### Apoptotic and hERG activity effects and hepatotoxicity of vitexicarpin

**FIGURE 6:**
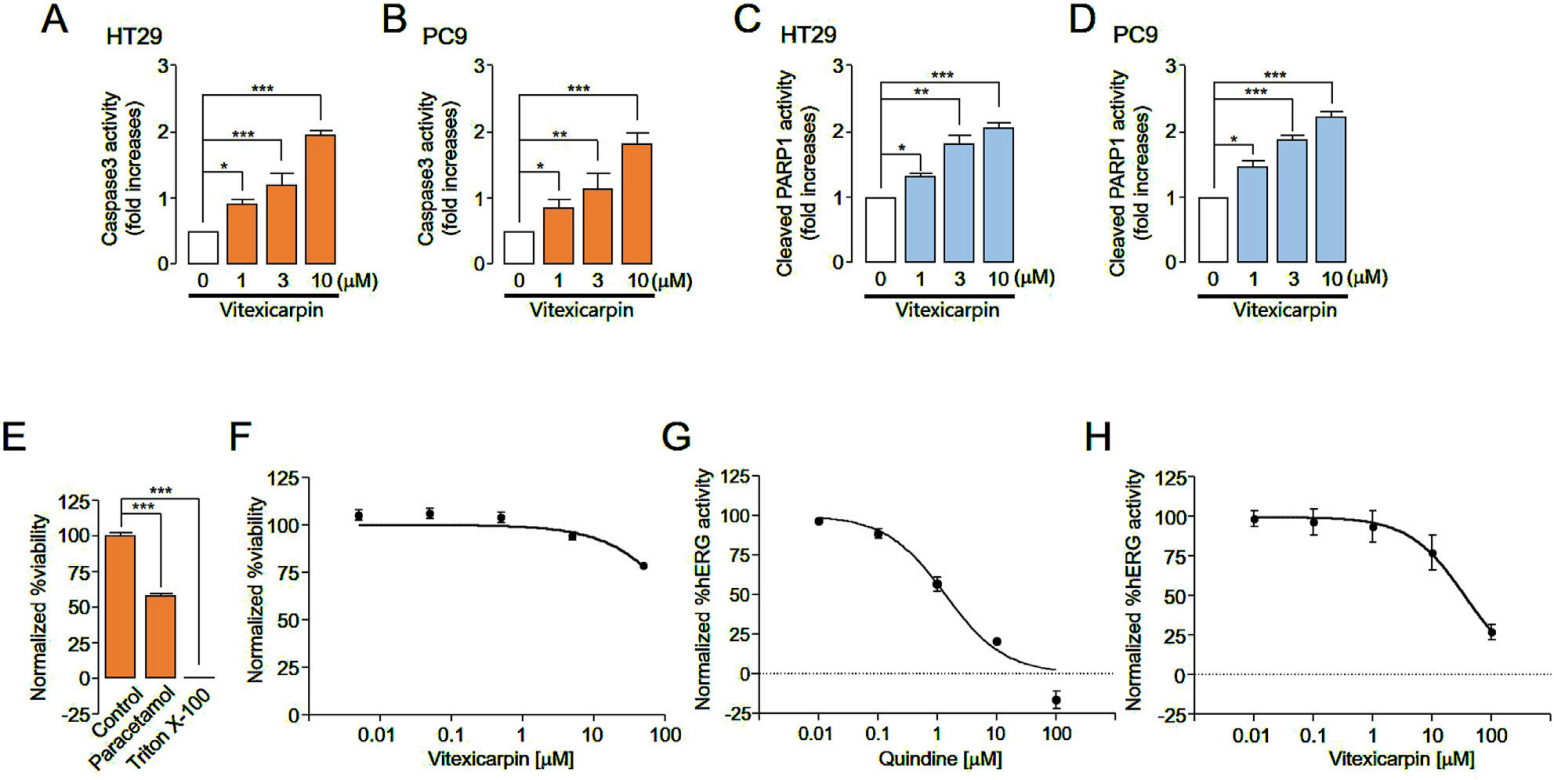
Evaluation of apoptosis, hERG channel activity inhibition, and liver cell viability following vitexicarpin and Ani9 treatment. **(A,B)** Caspase-3 activity in HT29 and PC9 cells cultured with vitexicarpin at the indicated concentrations for 24 h **(C,D)** Cleaved PARP1 levels in HT29 and PC9 cells incubated with the indicated concentrations of vitexicarpin for 24 h **(E,F)** Relative viability of HepG2 cells treated with the test compounds at five different concentrations (0.005, 0.05, 0.5, 5, and 50 μM). The viability data points were normalized considering the maximum viability obtained with 0.5% (v/v) dimethyl sulfoxide solvent treatment as 100% viability and the minimum viability obtained with 0.01% (v/v) Triton X-100 treatment as 0% viability. **(G,H)** hERG channel activity of HEK cells treated with the test compounds at five different concentrations (0, 0.01, 0.1, 1, 10, and 100 μM). Each data point was normalized using the baseline level of the hERG channel current (mean ± SD, *n* = 3–5). vitexicarpin. *p < 0.05, **p < 0.01, ***p < 0.001, Student’s unpaired *t*-test.

Since the pharmacological inhibition of ANO1 in various cancer cell types reportedly causes apoptosis (Ji et al., 2019; Fujimoto et al., 2017; Song et al., 2018), we evaluated the apoptotic effects of vitexicarpin in HT29 and PC9 cells. Vitexicarpin significantly increased caspase-3 activity and PARP-1 cleavage, two hallmarks of apoptosis (Figures 6A–D), and increased the sub-G1 population (Supplementary Figure S3). These results show that vitexicarpin exerts its anticancer effects by inducing apoptosis and inhibiting ANO1 expression.

To verify whether vitexicarpin is hepatotoxic, HepG2 cells were treated with vitexicarpin, and their viability was determined. Vitexicarpin up to 50 µM did not reduce liver cell viability compared with paracetamol (Figures 6E,F). In addition, the effects of vitexicarpin on hERG channel activity were tested in hERG channel-expressing HEK293 cells to confirm its potential application in cancer treatment. Vitexicarpin exhibited a low effect on hERG channel activity with an IC_50_ of 35.4 µM. In contrast, 10 μM quinidine (positive control) completely inhibited the hERG channel (Figures 6G,H).

## Discussion

Although the physiological roles of ANO1 are diverse, ranging from chloride ion secretion to oncogenesis (Liu et al., 2015; Seo et al., 2020a; b; Seo et al., 2016), targeting ANO1 channel function and diminishing its protein expression have shown therapeutic potential in various cancer cells (Park et al., 2023; Seo et al., 2020a; Seo et al., 2017). Recently, ANO1 has been identified as a promising therapeutic target for several malignancies, including colorectal cancer (CRC) and non-small cell lung cancer (NSCLC). However, no effective compounds have yet been discovered that target ANO1 for the treatment of CRC and NSCLC. (Bourreau et al., 2023; Ji et al., 2019; Fujimoto et al., 2017; Song et al., 2018). Consequently, there is an urgent need for novel molecules capable of inhibiting ANO1 channel function or downregulating ANO1 protein levels.

In our study, we identified vitexicarpin as a selective inhibitor of ANO1 channel function. Remarkably, vitexicarpin did not inhibit CFTR activity or alter ATP-induced calcium levels, indicating its specificity for ANO1 (Figures 1, 2). Vitexicarpin interacts with a known calcium-binding site in ANO1 (Figure 3). Although Ani9, another ANO1 inhibitor, also interacts with this site, vitexicarpin demonstrated a higher binding affinity (−49.9 kcal/mol) compared to Ani9 (−24 kcal/mol) (Supplementary Figure S2).

Current ANO1 inhibitors simultaneously inhibit ANO1 channels and reduce ANO1 protein levels, making it challenging to determine which function is directly responsible for the anticancer effect (Jeong et al., 2022). For example, Ani9 reduced ANO1 protein levels in HT29 cells but not in PC9 cells (Figure 4). Consequently, Ani9 did not affect PC9 cell viability; however, by reducing ANO1 protein levels, Ani9 decreased HT29 cell viability (Figures 4, 5). Thus, reducing ANO1 protein levels can influence cell viability.

The T790M mutation is a known mechanism of acquired resistance to first-generation epidermal growth factor receptor tyrosine kinase inhibitors (EGFR-TKIs), such as gefitinib, in NSCLC patients who are subsequently treated with the third-generation EGFR-TKI AZD9291 (osimertinib) (Leonetti et al., 2019; Roskoski, 2022). However, strategies to overcome acquired EGFR-TKI resistance in NSCLC patients without the T790M mutation are still lacking (Wang et al., 2023). In this study, we developed gefitinib-resistant PC9 cells through progressive exposure to increasing gefitinib concentrations over 11 months. Vitexicarpin effectively overcame gefitinib resistance at the cellular level and showed a favorable toxicity profile compared to synthetic inhibitors *in vitro*. Additionally, vitexicarpin exhibited substantial safety compared to synthetic compounds like osimertinib, suggesting its potential as a therapeutic candidate for gefitinib-resistant lung cancer (Figure 5). Furthermore, vitexicarpin induced apoptosis in both cancer cell lines and demonstrated low toxicity, indicating its promise as a safe and effective treatment option (Figure 6). Nevertheless, evaluating additional pharmacokinetic parameters such as absorption, distribution, metabolism, excretion, and toxicity in animal models is essential before considering human application.

In conclusion, cell-based high-throughput screening identified vitexicarpin’s anticancer potential. Vitexicarpin significantly inhibited ANO1 function and protein levels, reduced cancer cell viability, and induced apoptosis. Additionally, vitexicarpin inhibited growth of gefitinib-resistant cells in NSCLC. As a natural product with no hepatotoxicity or adverse cardiac effects, vitexicarpin holds promise as a valuable pharmacological tool in ANO1 inhibitor research and as a potential therapeutic for CRC and lung cancers, particularly in cases of resistance to existing anticancer drugs. Further research is needed to confirm whether vitexicarpin, a relatively non-toxic natural product, can overcome resistance to current anticancer therapies in these specific cancer types.

## Data Availability

The original contributions presented in the study are included in the article/Supplementary Material, further inquiries can be directed to the corresponding authors.
