# Supplementary figures and images for: Vitexicarpin suppresses colorectal and non-small cell lung cancer via selective inhibition of Anoctamin 1
Source: Front Pharmacol. 2025 May 30;16:1557193. doi: 10.3389/fphar.2025.1557193 (PMC12162337; doi:10.3389/fphar.2025.1557193)

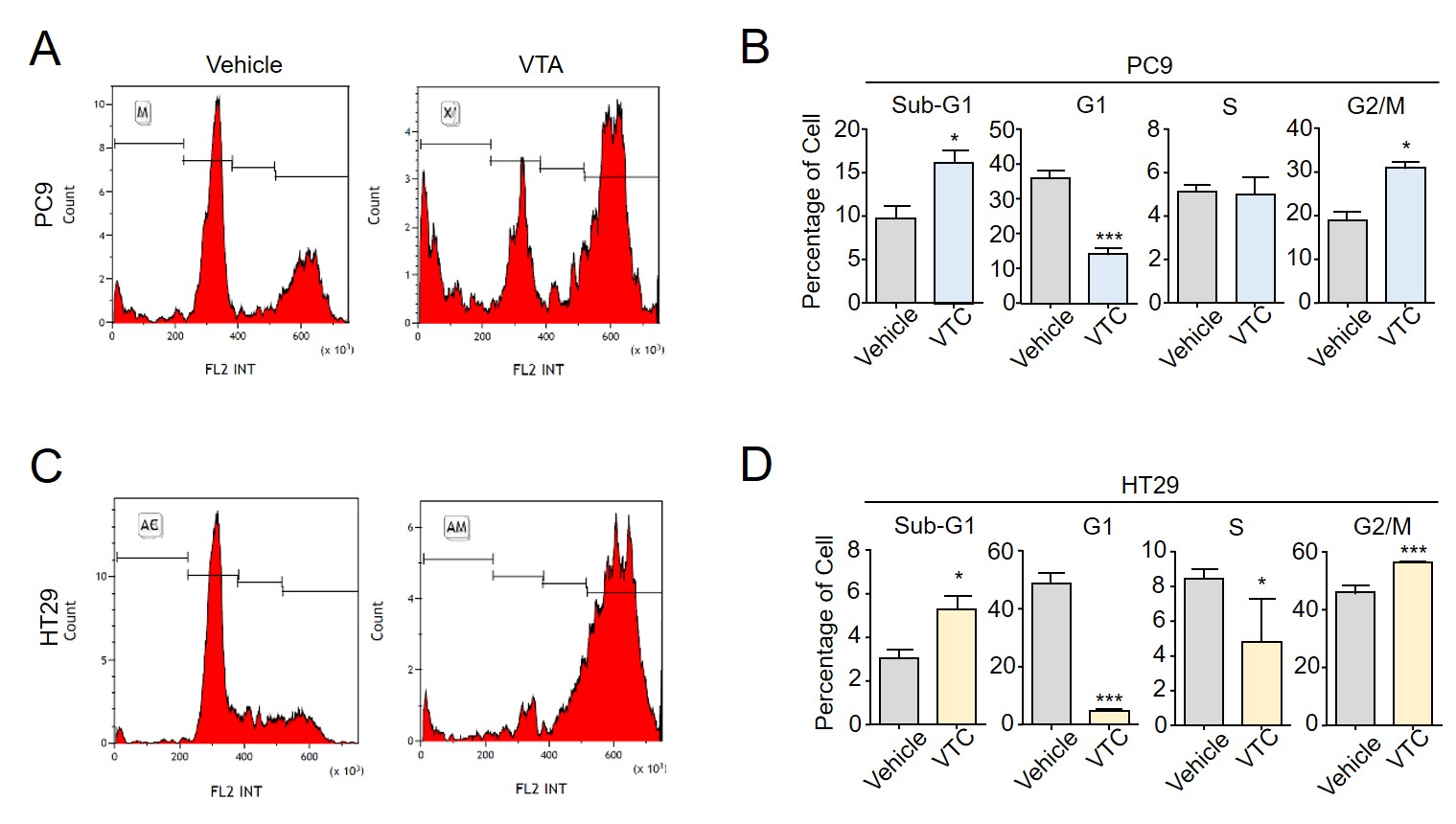

Supplement: Supplementary file 1 [file Image3.jpeg]

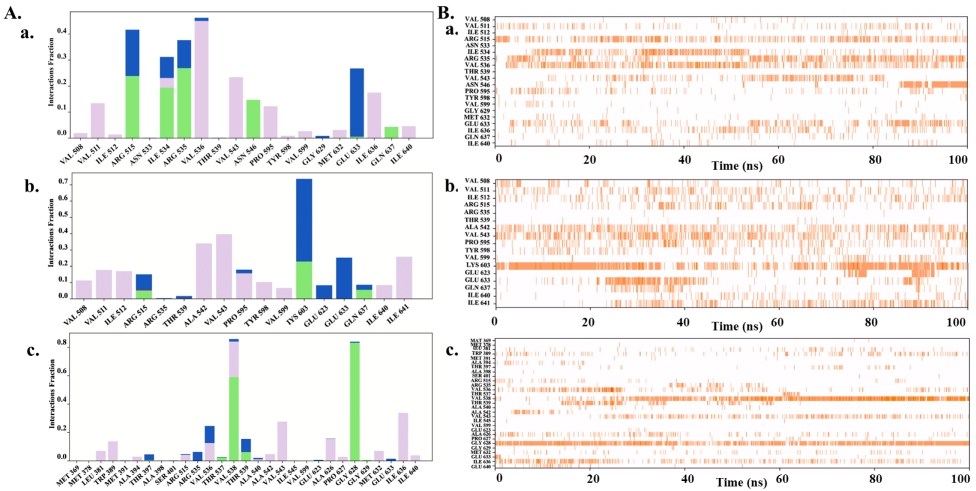

Supplement: Supplementary file 2 [file Image1.jpeg]

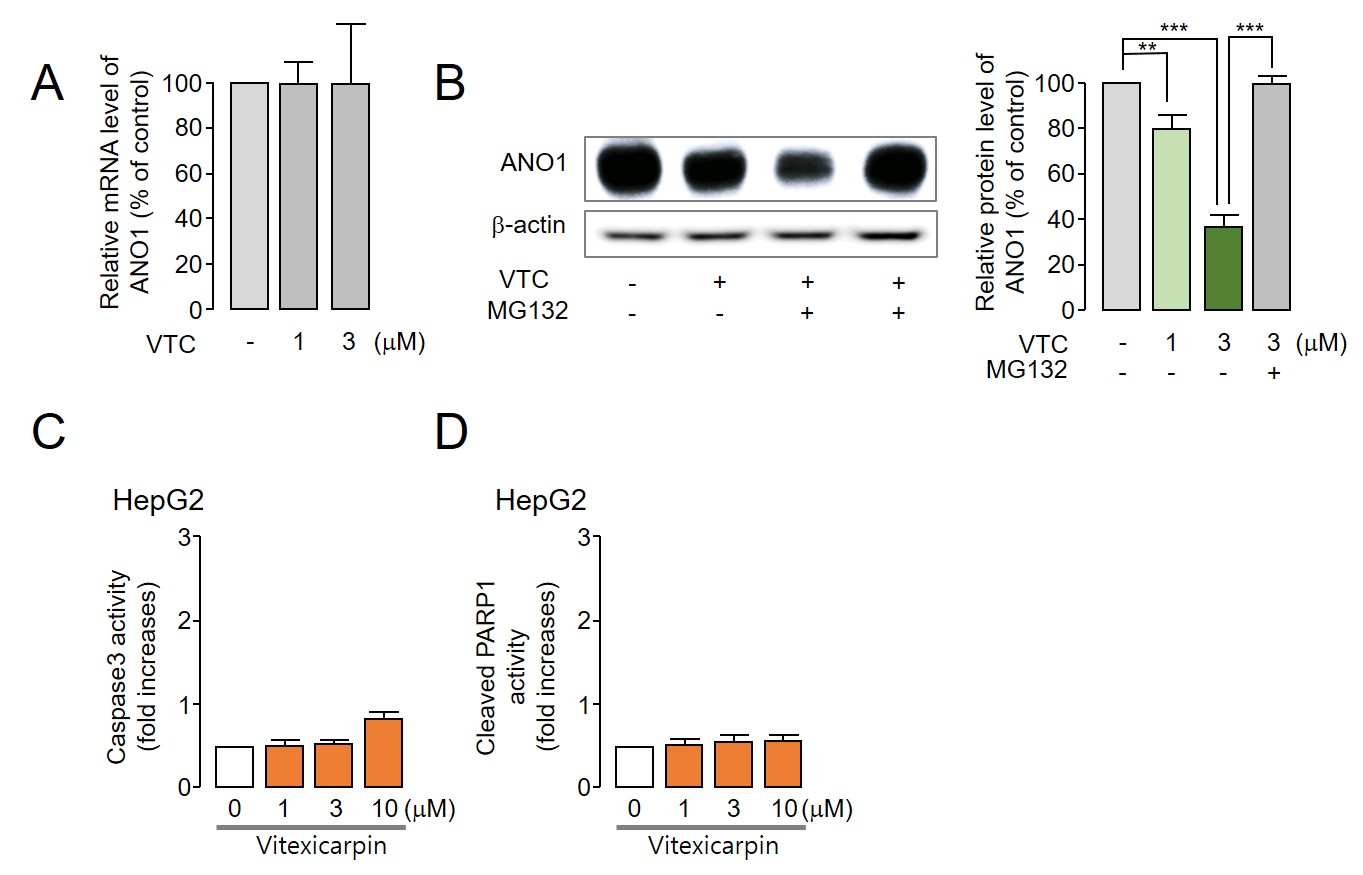

Supplement: Supplementary file 3 [file Image4.jpeg]

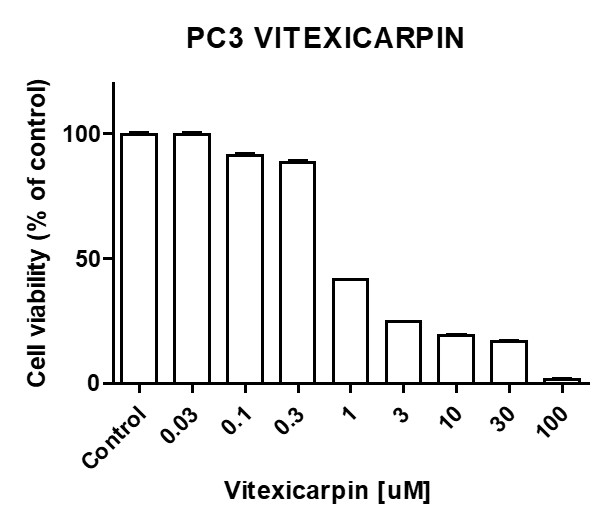

Supplement: Supplementary file 4 [file Image2.jpeg]

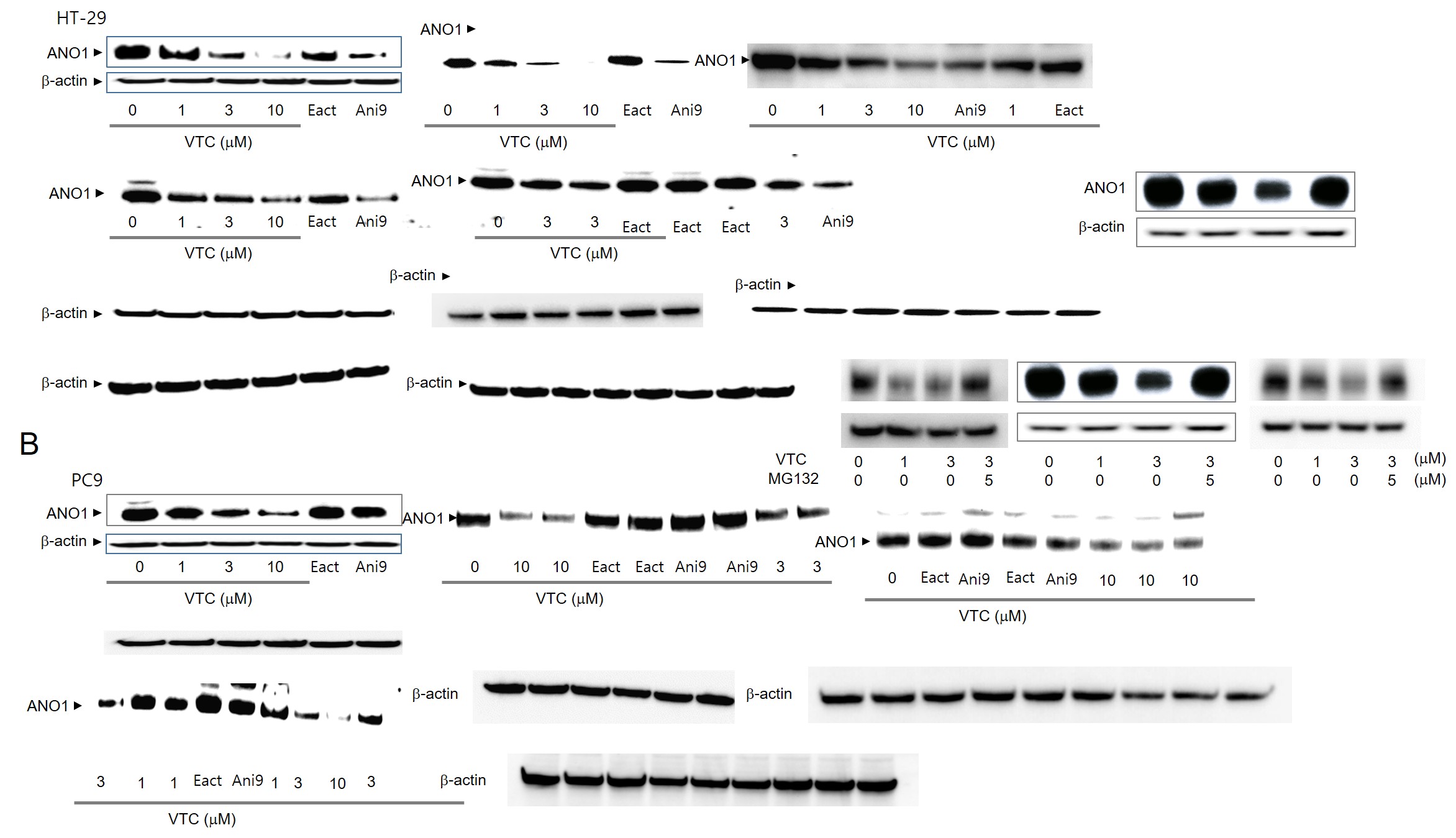

Supplement: Supplementary file 5 [file Image5.jpeg]
