# Supplementary material for: Vitexicarpin suppresses colorectal and non-small cell lung cancer via selective inhibition of Anoctamin 1
Source: Front Pharmacol. 2025 May 30;16:1557193. doi: 10.3389/fphar.2025.1557193 (PMC12162337; doi:10.3389/fphar.2025.1557193)
Supplement: Supplementary file 6 [file DataSheet1.docx]

Vitexicarpin demonstrates anticancer potential by inhibiting anoctamin 1 in colorectal and non-small cell lung cancers

Yohan Seo^1,2^*, Sion Lee^2^, Raju Das^3^, Sung Baek Jeong^2^, Chul Soon Park^1^, Minuk Kim^4^, Deok Kyu Yoon^4^, Armin Sultana^3^_,_ Kantu Das^5^, Jae-Eon Lee^6^, Yong Hyun Jeon^6^, Phan Thi Thanh Huong^7,^ Nguyen Xuan Nhiem^7,^ Joohan Woo^3,8^*

**Supplementary Table S1A. Docking and binding free energy (MM-GBSA) scores for the selected compounds.**

| **Compound Name** | **ANO1**  **PDB ID: 7ZK3** | |
| --- | --- | --- |
|  | **XP Docking Score (Kcal/mol)** | **MM-GBSA (Kcal/mol)** |
| Ani 9 | -6.686 | -24.00 |
| Eact | -5.339 | -52.71 |
| Vitexicarpin | -6.675 | -49.90 |

**Supplementary Table S1B. Docking interaction patterns of ANO1 (7ZK3) with selected compounds.**

| **Compound Name** | **Hydrogen bond (length in Å)** | **Hydrophobic bond (length in Å)** | **π-cation** | **Halogen bond (length in Å)** |
| --- | --- | --- | --- | --- |
| Ani 9 | ARG^515^ (3.42)  ARG^515^ (2.67)  LYS^603^(2.25) | VAL^511^(3.43)  ILE^512^ (3.73)  VAL^543^(3.19)  ASN^546^ (3.83)  LEU^547^(3.95)  PRO^595^(3.45)  TYR^598^(3.30)  VAL^599^(3.99)  GLN^637^(3.56)  ILE^640^ (3.80)  ILE^641^ (3.36) | TYR^867^(4.83) | **-----------** |
| Eact | ARG^515^ (2.77)  ARG^535^ (2.95) | ARG^535^ (3.89)  THR^539^ (3.99)  VAL^599^(3.78)  GLU^633^(3.90)  ILE^636^ (3.51) | **-----------** | **-----------** |
| Vitexicarpin | ARG^515^ (3.21)  ARG^535^ (2.75)  THR^539^(3.53)  GLU^633^(1.76) | VAL^599^(3.79)  THR^539^(3.91)  GLU^633^(3.36)  ILE^636^(3.61)  ILE^636^(3.70) | **-----------** | **-----------** |

**
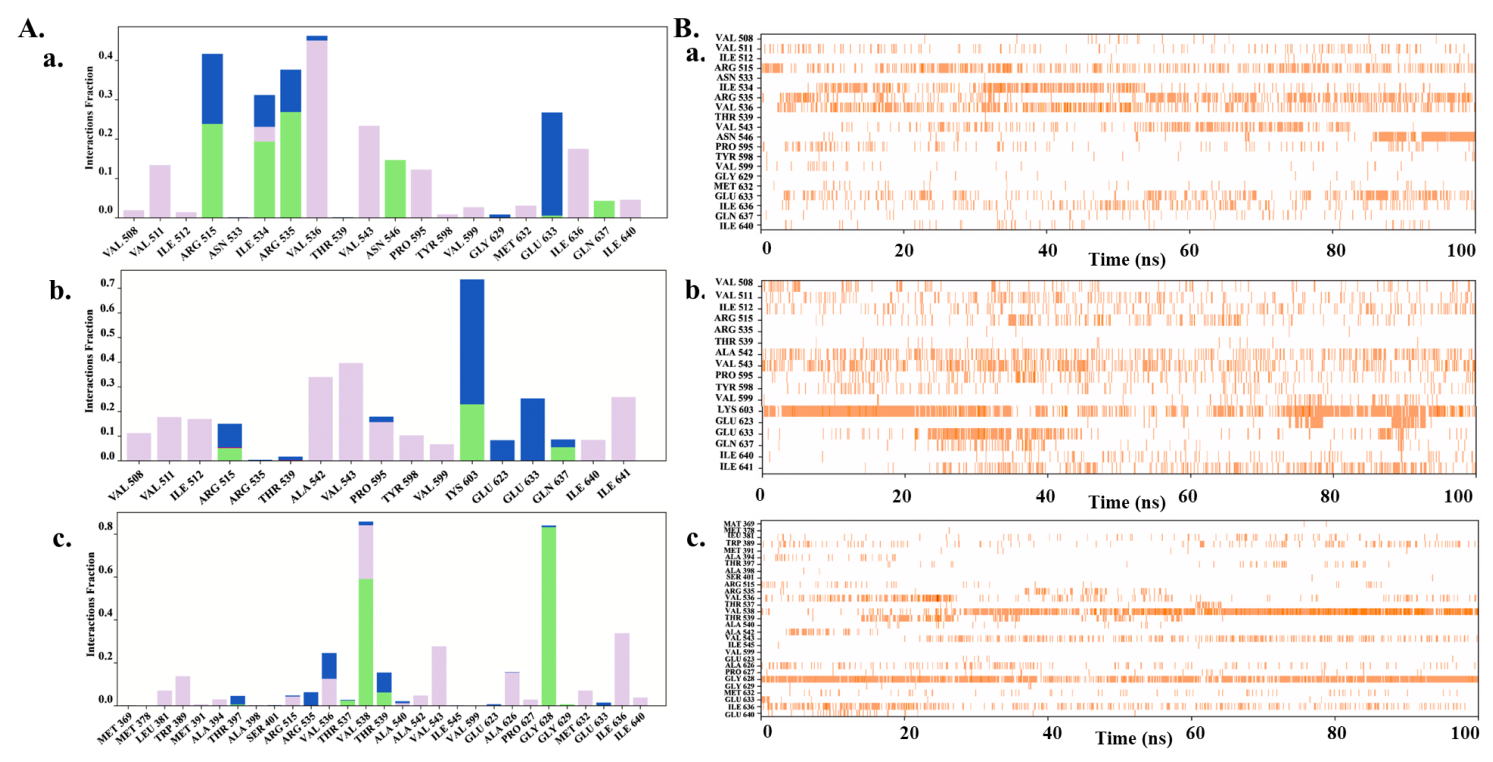
**

Supplementary figure1. Total protein-ligand interaction pattern during 100 ns dynamics simulation time. A) Total protein-ligand of a. Vitexicarpin, b. Ani 9, and c. Eact. B) The residues involve in ligand interaction of a. vitexicarpin, b. Ani 9, and c. Eact. Dark brown color represents the more than one interaction per trajectory.


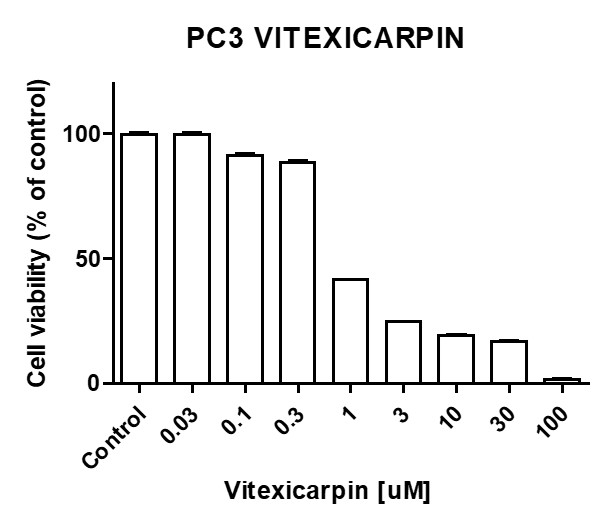


Supplementary figure2. Effects of vitexicarpin on PC3 cell viability.


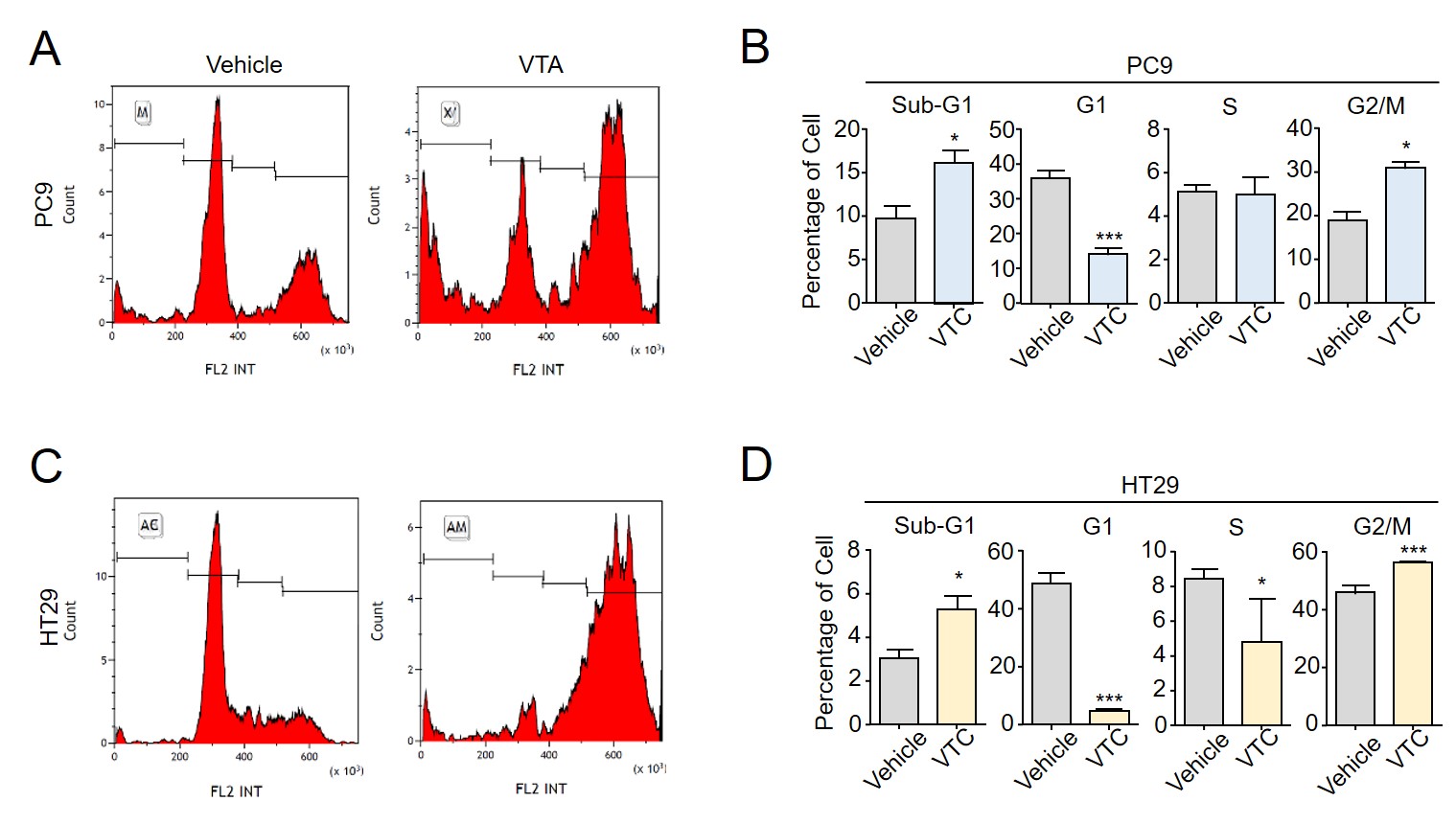


**Supplementary Figure 3.** Analysis of cell cycle distribution in PC-9 and HT29 cells treated with Vitexicarpin. (A–D) PC-9 or HT29 cells were treated with 3 μM Vitexicarpin for 24 h. Cell cycle distribution was analyzed by propidium iodide (PI) staining followed by flow cytometry. Data are presented as mean ± SD from three independent experiments (n = 3). Statistical significance was assessed using Student’s unpaired t-test (p < 0.05, p < 0.01, *p < 0.001).


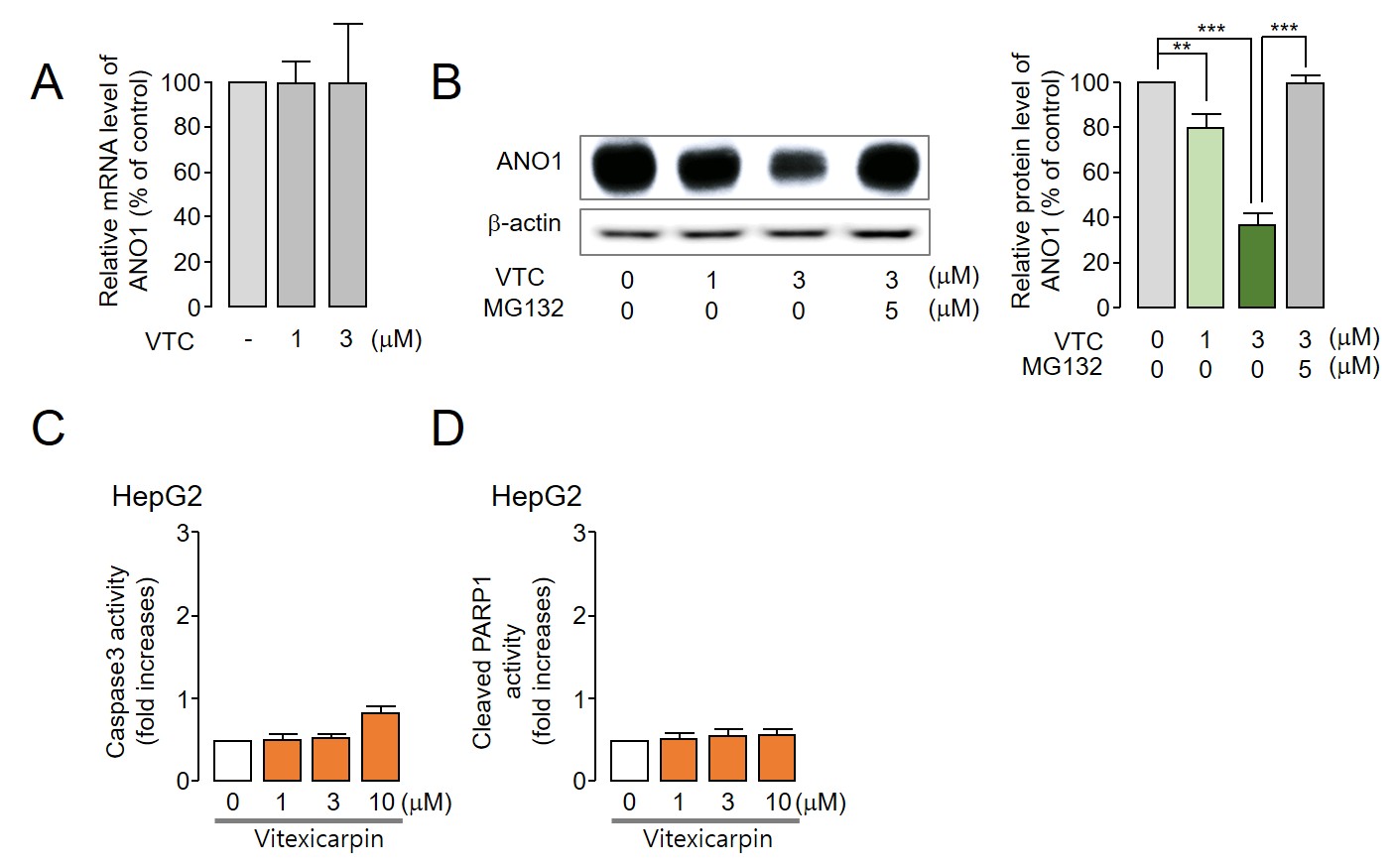


**Supplementary Figure 4**. Effects of Vitexicarpin on ANO1 expression in HT29 cells and apoptosis markers in HepG2 cells.
(A) Quantitative real-time RT-PCR analysis of ANO1 mRNA levels in HT29 cells treated with 1 or 3 μM Vitexicarpin for 24 h. β-actin was used as the loading control. (B) Western blot analysis of ANO1 protein expression in HT29 cells following treatment with 1 or 3 μM Vitexicarpin, with or without MG132 (5 μM, 24 h pre-treatment). The right panel shows densitometric quantification of ANO1 expression normalized to β-actin. (C) Caspase-3 activity in HepG2 cells treated with Vitexicarpin at concentrations of 1, 3, or 10 μM for 24 h. (D) Cleaved PARP1 levels in HepG2 cells treated with the indicated concentrations of Vitexicarpin (1, 3, and 10 μM) for 24 h. All data are presented as mean ± SD from three independent experiments (n = 3).
